# Associations between socioeconomic status and physical activity: A cross-sectional analysis of Chinese children and adolescents

**DOI:** 10.3389/fpsyg.2022.904506

**Published:** 2022-09-01

**Authors:** Youzhi Ke, Lijuan Shi, Lingqun Peng, Sitong Chen, Jintao Hong, Yang Liu

**Affiliations:** ^1^School of Physical Education, Shanghai University of Sport, Shanghai, China; ^2^Department of Comprehensive Education and Preschool Education, Shanghai Teacher Training Center, Shanghai, China; ^3^School of Physical Education, Shanghai University of Electric Power, Shanghai, China; ^4^Institute for Health and Sport, Victoria University, Melbourne, VIC, Australia; ^5^Shanghai Research Institute of Sports Science, Shanghai, China; ^6^Shanghai Research Center for Physical Fitness and Health of Children and Adolescents, Shanghai, China

**Keywords:** social inequalities, physical activity, health promotion, children, youth

## Abstract

**Objectives:**

Although socioeconomic status (SES) has been shown to be an important determinant of physical activity (PA) in adults, the association in children and adolescents remains less consistent based on evidence from western developed countries. The purpose of this study, therefore, is to investigate associations between SES and PA among Chinese children and adolescents.

**Methods:**

A cross-sectional study was conducted with a self-reported questionnaire in China. The multi-stage stratified cluster sampling method was used, and 2,955 children and adolescents (8–17 years old, 53.4% girls) were enrolled in this study. SES was assessed by measuring parental education levels, perceived family wealth, and Family Affluence Scale II (FAS II). PA was assessed by reliable and valid items from the Health Behavior School-aged Children Surveillance questionnaires. Descriptive statistics were used to report sample characteristics, including demographic characteristics, levels of PA, and SES. The Chi-square test was applied to analyze the differences of PA in gender and school period. Binary logistic regression was used to analyze the association between SES and PA among children and adolescents.

**Results:**

Overall, 10.4% of children and adolescents accumulated moderate-to-vigorous physical activity (MVPA) at least 60 min per day. Boys were more physically active than girls (14.1% vs. 7.2%, *p* < 0.001). Higher socioeconomic status was positively associated with higher physical activity levels among children and adolescents, especially using FAS and mother education level as SES measures. The association between SES and PA varied by gender and grade group, and the effects of SES are also different on weekdays and weekends.

**Conclusion:**

This study found socioeconomic disparities in PA among Chinese children and adolescents, and showed the importance of targeting children and adolescents with low SES intervention priority. Based on these research findings, it is suggested that SES, especially for FAS and mother education level, should be considered when designing and implementing the promotion of regular PA in children and adolescents. Health policymakers may use this information to develop interventions to reduce health inequalities among children and adolescents in the future.

## Introduction

The public health challenge of insufficient physical activity (PA) among children and adolescents has been well documented (Music Milanovic et al., 2021). Not getting enough physical activity can raise the risk of developing many non-communicable diseases, such as heart disease, diabetes, and cancers (Lona et al., 2021). In addition, evidence supporting the benefits of physical activity and exercise to physical and mental health continues to accumulate (Kemel et al., 2021). A number of studies have shown that physical activity can improve emotional and cognitive abilities and reduce depression and anxiety symptoms (Borland et al., 2022), also enhance the experience of well-being in children and adolescents (Lema-Gomez et al., 2021). Early childhood is a crucial period during which children have sufficient opportunities to develop healthy habits that could be maintained from adolescence to adulthood (Music Milanovic et al., 2021). Despite the widely known benefits of physical activity, many young people do not meet recommended levels of physical activity. The World Health Organization (WHO) recommends that children aged 5–17 years accumulate at least 60 min of moderate- to -vigorous PA (MVPA) per day (World Health Organization, 2020).Globally, more than 80% of students aged 11–17 years do not meet the recommended PA level set by the WHO, especially students in the high-income countries in the Asia-Pacific region (Guthold et al., 2020). In China, only 13.1% of children and adolescents (9–17 years old) meet the recommended 60-min MVPA per day (Liu et al., 2019). According China Report Card, boys (14.5%) were more active than girls (11.7%). The rates of meeting the PA guidelines decreased gradually with age from primary school (18.9%) to secondary school (11.9%), and finally to upper secondary school (8.0%; Liu et al., 2019).Furthermore, the time trend study also shows that from 2004 to 2015, the total physical activity of Chinese children and adolescents aged 6–17 showed a downward trend (Yang et al., 2021).

Researchers studying the Chinese population have found that the lack of physical activity of Chinese children is related to the environment, personal and family or behavioral fields (Tian et al., 2021). Despite these results, data on exploring the relationship between SES and PA in children and adolescents in Asian countries, especially in China, is surprisingly scarce. China is the largest country in the world by population, has the largest economy, and is experiencing a technology boom (Central Intelligence Agency, 2018; International Monetary Fund, 2018). As technology advances and lifestyles change, the increasing number of various screen-based learning devices and the ease of transportation have reduced PA for children and young people (Liu et al., 2016). Hence, focusing efforts on controllable factors to increase PA in high-risk groups is critical (Tandon et al., 2012).To address these health issues, it is needed to gain a better understanding of determinants of PA in children and adolescents and in turn develop effective PA promotion interventions. PA participation in children and adolescents appears to be influenced by multiple factors, including personal, environmental, social, and psychological (Sallis et al., 1992). Identifying socioeconomic determinants of PA is particularly important as these informative messages can be used to inform equity policies to reduce health inequalities (Music Milanovic et al., 2021).

Socioeconomic status (SES) was defined as “the relative position of a family or individual on a hierarchical social structure, based on their access to or control over wealth, prestige and power” (Mueller et al., 1981). Researchers are currently studying two theoretical models that explain key influencing factors in the early stages of human development and the continuous impact of SES in the life cycle. The latency model highlights the potential impact of early experiences on all aspects of life, while the path model emphasizes the cumulative and interactive effects of socioeconomic and psychosocial factors during a lifetime (Keating and Hertzman, 2000). Currently, more research is focusing on pathway modeling aspects of the potential compensatory impact of cumulative advantage in an effort to understand health outcomes. For example, social causation theory suggests that health risk behavior or ill-health is more common among people in lower social class, which emphasizes the role of SES in the distribution of health and well-being (Goldman, 2001). More specifically, differences in SES indicate differences in the ability to access resources, affecting an individual’s ability to engage in varied healthy (or unhealthy) behaviors, then with a more profound impact on PA (Gidlow et al., 2006). PA in children and adolescents has been found to be associated with SES in many studies, while those findings were inconsistent (Stalsberg and Pedersen, 2010). For example, no statistical significance was observed between MVPA and SES when comparing children from high SES with those counterparts from low SES in a study conducted in Scotland (Donnelly et al., 2021). However, data from the Health Behavior in School-aged Children 2017/2018 study revealed that PA participation levels were lower among adolescents from less affluent families (Inchley et al., 2020). Furthermore, a national survey in Vietnam demonstrated that individuals in low SES groups tended to be more physically active than those in high SES groups (Vu et al., 2020). The role of SES as a predictor of PA in children and adolescents is controversial, and whether there are variations in developing countries remains to be clarified. Thus, a significant need exists to replicate these studies in different countries to improve the generalizability of previous evidence (Ford et al., 1991).

Moreover, the research on the association between SES and PA is further complicated by the multifaceted nature of SES and the lack of standardized definitions and measurements (Music Milanovic et al., 2021). Additionally, coupled with the difficulty in accurately assessing PA in a standardized manner, it leads to variability in the measurement method (O’Donoghue et al., 2018). The most widely used indicators of SES have been education, income, and occupation (Goodman et al., 2001; Galobardes et al., 2007). Overall, parental education seems to be the strongest and most consistent predictor of physical activity in children and adolescents (Winkleby et al., 1992; Galobardes et al., 2006), occupation may not be a useful measure for teen mothers (Ensminger et al., 2000; Daly et al., 2002), and nonresponse for income is often higher than nonresponse rates for other variables (Roemer, 2000). In recent years, material wealth and perceived family wealth have also been commonly used as measures of SES (Music Milanovic et al., 2021). Meanwhile, the indicators suitable for evaluating the SES are significantly varied due to differences in the regional environment, culture, and economic development, and the choice of SES measure(s) should ideally be informed by consideration of the specific research question and the proposed mechanisms linking SES to the outcome (Galobardes et al., 2006). Therefore, this study chose parental education, material wealth and perceived family wealth as measures of SES, taking into account the actual situation in China and the applicability of the indicators.

To the best of our knowledge, the association between SES and PA in children and adolescents has been extensively studied in western developed countries, while evidence in developing countries is limited. In order to successfully promote physical activity among children and adolescents in different regions, more information is needed on the impact of socioeconomic factors on PA levels among children and adolescents (Kantomaa et al., 2007). Therefore, this study aims to investigate associations between SES and PA among children and adolescents aged 8 to 17 years in China. In particular, this study examined whether SES, as indicated by parental education, material wealth and perceived family wealth, would be associated with PA of children and adolescents in China. It was hypothesized that children and adolescents in low SES would be less physically active than children and adolescents in high SES.

## Materials and methods

### Study design and sampling procedure

This study was a cross-sectional school survey conducted from September to December 2019 in Jiangsu, Anhui, and Zhejiang provinces, as well as Shanghai municipality, China. Multistage sampling design to survey PA level of children and adolescents in primary, junior middle, and junior high schools. Since students below the Grade 3 were not considered capable of understanding the questionnaire, we only included students in Grade 3 to 12 this study. In the first stage, a total of 34 primary schools, junior middle schools, and high schools were selected from Jiangsu, Anhui, Zhejiang and Shanghai. The second stage, the random cluster sampling method was conducted to select classes in the target grade (3-12th) within the schools.

### Participants

Participants were 3,368 children from primary schools (Grades 3–6, 8–11 years old), junior middle schools (Grades 7–9, 12–14 years old), and high schools (Grades 10–12, 15–17 years old,), with students ranging in age from 8 to17 years old (representing youth with an average ages of 13.3 ± 2.5 years). In response, 2,955 students (response rate = 87.7%) completed the self-reported questionnaire. There were two reasons why students did not complete the questionnaires: (1) Some students were absent due to illness and did not complete it; (2) Due to an academic test during data collection, some students were not able to complete the questionnaire; Several questionnaires were excluded due to: (1) Missing or abnormal values for key variables (e.g., gender, grade); (2) Missing or abnormal values were found for dependent variables (e.g., MVPA); (3) Missing or abnormal values were found for independent variables (e.g., SES).

### Procedures

The study protocol and procedure were approved by the Institutional Review Board (IRB) of the Shanghai University of Sport (SUS), and the permission to conduct the study was obtained from the teachers and principals of the participating schools. The IRB of SUS approved that verbal consent is sufficient to conduct this study due to the fact that none of the survey items related to personal ethic issues. All children and adolescents involved in the study, and their parents or guardians, were specifically advised that participation was completely voluntary. Verbal informed consent was obtained from all parents or guardians, and positive assent was obtained verbally from all children and adolescents prior to data collection. Trained research assistants implemented the survey according to a standardized survey administration protocol during regular school hours. The survey was completed on paper in a classroom setting. Students were instructed on how to fill out the survey and were provided ample time for questions. Data from the survey were collected and analyzed anonymously.

### Measurements

All measures used in this study were based on self-reports from surveys, children and adolescents were required to report their demographic information, including gender (1 = male, 2 = female), grade (Grades 3 to 12), ethnicity (Han or others). For children younger than 10 years old, questionnaires were completed with the assistance of trained research assistants. Details of each measure in this study are described below.

#### Assessment of physical activity

MVPA was assessed using two adapted items of the HBSC questionnaire (Prochaska et al., 2001; Inchley et al., 2016; Sigmund et al., 2018). Those items have been confirmed as feasible and reliable measures of PA for Chinese children and adolescents (reliability coefficient = 0.82; Liu et al., 2010). To calculate MVPA, each student answered the following question: “In the past week, how many days did you participate in moderate-to-vigorous physical activity for at least 60 min from Monday to Friday? (1 = 0 days; 2 = 1 day; 3 = 2 days; 4 = 3 days; 5 = 4 days; 6 = 5 days); From Saturday to Sunday, how many days did you participate in moderate-to-vigorous physical activity for at least 60 min? (1 = 0 days; 2 = 1 day; 3 = 2 days).” So that students would have a better understanding of MVPA, it was explained as “any kind of physical activity increasing your heart rate and breathing frequencies during a period (including physical education, physical exercising, sports training, and various regular daily activities, such as brisk walking, hiking, and excursion).” According to the WHO physical activity recommendation standard (World Health Organization, 2020) and the Canadian 24-Hour Movement Guideline (Tremblay et al., 2016), for our primary outcome we defined a student as meeting current guidelines if he or she was active at least 60 min on each of the last 7 days (yes/no; Krist et al., 2017).

#### Assessment of socioeconomic status

Three individual measures of SES were examined from the Canadian HBSC: a measure of material wealth (Family Affluence Scale II), a measure of perceived family wealth and parental education. The FAS II has been used extensively in the HBSC study in the past decade to examine and describe socioeconomic inequalities in relation to adolescent health outcomes (Liu et al., 2012). FAS II consists of 4 questions, taking the range (0–9) and then divided into 3 categories, showed a reliable and valid SES measure for children and adolescents in China (Cronbach’s alpha = 0.58; ICC > 0.75; Liu et al., 2012).

The 3-point Family Affluence Scale II (low, medium, or high) was developed on the basis of fours measures of material family wealth, as reported by the students (car ownership, bedroom sharing, holiday travel, and computer ownership; Currie et al., 2008). Do you have a car at home? (No = 0; one = 1; yes, more than two = 2); How many computers do you have at home? (No = 0; one set =1; two sets = 2; more than two sets = 3); in the past year, how many times did you and your family travel during the holidays? (No = 0; once = 1; twice = 2; more than twice = 3); do you have your own room? (No = 0; Yes = 1). In the analysis process, FAS is divided into three categories (“low,” “medium,” and “high”). The FAS category corresponds to the tertile of the total score (“low” = 0–2; “medium” = 3–5, “High” = 6–9).

Perceived family wealth was designed to measure the students’ perceptions of their family’s socioeconomic circumstances. This variable was based on the question “How well off do you think your family is?” and the following response categories: very well off, quite well off, average, not very well off, not at all well off. In the process of analysis, perceived family wealth was divided into three categories, low economic level (not very well off, not at all well off), medium economic level (average), and high economic level (very well off, quite well off).

Parental education levels were determined based on reported data in this study and categorized them into seven groups: (1) Below elementary school, (2) elementary school, (3) junior high school, (4) high school or technical secondary school, (5) college, (6) undergraduate, (7) graduate and above. In the process of analysis, the parental education background is divided into three categories, low education level (below elementary school, elementary school, junior high school), middle education level (high school or technical secondary school, junior college), and high education level (undergraduate, postgraduate and above).

### Statistical analyses

The collected questionnaires were sorted in Excel and analyzed with SPSS 24 software. The missing cases and abnormal values were removed. According to the aims of this study, variables of grade (age), gender and PA were included in the statistical analysis. According to the Chinese school-aged population of children in the current school education system, age groups are divided according to different grade (i.e., primary, junior middle, and junior high schools). Use descriptive statistics to analyze the basic situation of the survey object (demographic information, PA and SES), continuous variables were expressed as mean and standard deviation (mean ± standard deviation), and categorical variables were expressed as numbers (*n*) and percentages (%). Between-group differences in demographic variables were tested with a chi-square test for categorical variables. Prevalence percentage of meeting MVPA recommendations were calculated by: sex (boys and girls) and school grades [primary schools (Grades 3–6, 8–11 years old); junior middle schools (Grades 7–9, 12–14 years old); and high schools (Grades 10–12, 15–17 years old)] on whole week, weekdays and weekends. Binary logistic regression analyze the association of PA and SES adjusted for age. Logistic regression analysis results were presented as an odds ratio (OR) with 95% confidence interval (CI). *p* ≤ 0.05 is statistically significant.

## Results

The descriptive characteristics of the samples in this study are shown in Table 1. The survey finally included 2,955 people in the sample, including 1,378 boys (46.6%) and 1,577 girls (53.4%), with an average age of 13.36 ± 2.46 years (13.08 ± 2.43 for boys and 13.01 ± 2.47 for girls, *p* < 0.001) The proportions of the sample across the 3 grade groups were 17.9% (primary school: boys 19.5%, girls 16.4%); 61.2% (junior school: boys 67.8%, girls 55.5%); and 20.9% (high school: boys 12.7%, girls 28.1%), respectively. There was a statistically significant gender difference among grade groups (*p* < 0.001). Most participants were identified as Han ethnicity (96.9%), there was no significant difference among age groups (*p* > 0.05).The majority of participants’ fathers and mothers had low education levels (41.0% and 47.7%, *p* > 0.05), respectively. About 57.5% of participants had middle perceived family wealth (55.1% for boys, 59.5% for girls, *p* < 0.05), and 46.3% of participants with high FAS (46.7% for boys, 46.0% for girls, *p* > 0.05).

**Table 1:** The characteristics of the samples.

Category	Overall (2,955)	Boys (1,378)	Girls (1,577)	*P*
Age (years), M ± SD	13.36 ± 2.46	13.08 ± 2.43	13.01 ± 2.47	<0.001
Grade groups, *n* (%)				<0.001
Primary school	527 (17.9)	269 (19.5)	258 (16.4)	
Junior middle school	1,809 (61.2)	934 (67.8)	875 (55.5)	
High school	619 (20.9)	175 (12.7)	444 (28.1)	
Ethnicity, *n* (%)				0.071
Han	2,862 (96.9)	1,399 (97.2)	1,523 (96.6)	
Others	93 (3.1)	39 (2.8)	54 (3.4)	
**SES, *n* (%)**				
Father education level				0.345
Low	1,211 (41.0)	546 (39.6)	665 (42.2)	
Middle	1,021 (34.6)	483 (35.1)	538 (34.1)	
High	723 (24.4)	349 (25.3)	374 (23.7)	
Mother education level				0.113
Low	1,409 (47.7)	629 (45.6)	780 (49.5)	
Middle	899 (30.4)	433 (31.4)	466 (29.5)	
High	647 (21.9)	316 (23.0)	331 (21.0)	
Perceived family wealth				0.002
Low	333 (11.3)	143 (10.4)	190 (12.1)	
Middle	1,699 (57.5)	760 (55.1)	939 (59.5)	
High	923 (31.2)	475 (34.5)	448 (28.4)	
FAS II				0.71
Low	378 (12.8)	181 (13.2)	197 (12.5)	
Middle	1,208 (40.9)	553 (40.1)	655 (41.5)	
High	1,369 (46.3)	644 (46.7)	725 (46.0)	

M, Means; SD, standard deviation; SES, socioeconomic status; FAS II, Family Affluence Scale II. Primary school, 8–11 years old; Junior middle school, 12–14 years old; High school, 15–17 years old.

Table 2 presents the prevalence of participants meeting PA guidelines. The percentage meeting PA guidelines on whole week is 10.4%, and boys are higher than girls (14.1% vs. 7.2%, *p* < 0.001). The proportions of meeting PA guidelines on weekdays and weekends were 22.3% and 25.1%, respectively, and boys were higher than girls (*p* < 0.001). The percentages of meeting the PA guidelines in the three grade groups were different (primary school: 19.5%; junior school: 9.4%; high school: 5.5%, *p* < 0.001).

**Table 2:** The prevalence of meeting the PA guidelines.

Category	Whole week^a^^,^^b^, *n* (%)		Weekdays^a^^,^^b^, *n* (%)		Weekends^a^^,^^b^, *n* (%)	
	Not meet	Meet	Not meet	Meet	Not meet	Meet
Total	2,648(89.6)	307(10.4)	2,295(77.7)	660(22.3)	2,213(74.9)	742(25.1)
**Gender**						
Boys	1,184(85.9)	194(14.1)	1,009(73.2)	369(26.8)	951(69.0)	427(31.0)
Girls	1,464(92.8)	113(7.2)	1,286(81.5)	291(18.5)	1,262(80.0)	315(20.0)
**Grade groups**						
Primary school	424(80.5)	103(19.5)	373(70.8)	154(29.2)	289(54.8)	238(45.2)
Junior middle school	1,639(90.6)	170(9.4)	1,379(76.2)	430(23.8)	1,388(76.7)	421(23.3)
High school	585(94.5)	34(5.5)	543(87.7)	76(12.3)	536(86.6)	83(13.4)

PA, physical activity.

a denotes significant gender difference at *p* < 0.001.

b denotes significant grade group difference at *p* < 0.001.

Primary school, 8–11 years old; Junior middle school, 12–14 years old; High school, 15–17 years old.

**Figure 1:**
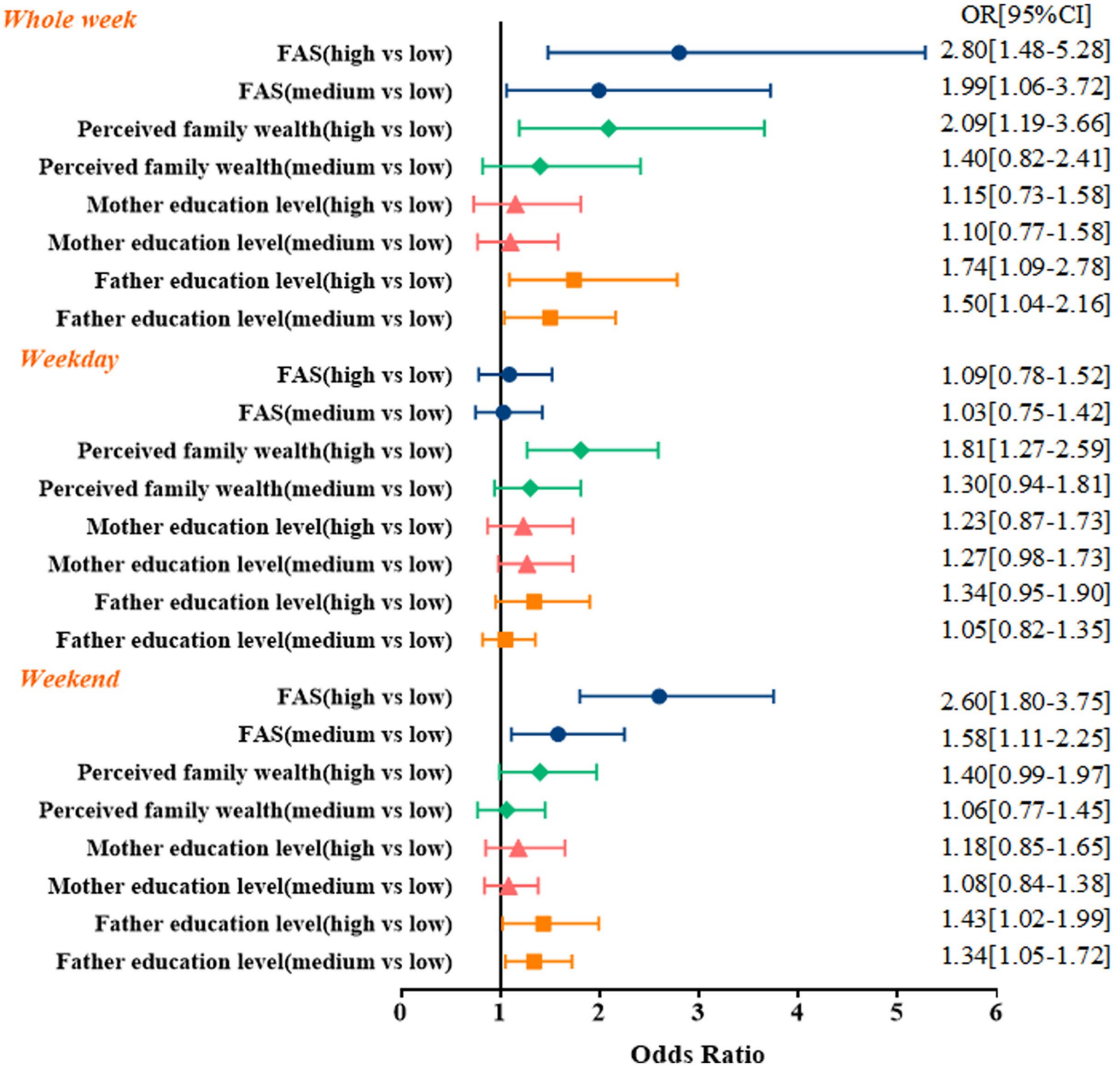
Regression analysis of socioeconomic status and physical activity.

The associations between SES and the prevalence of meeting the PA guidelines are presented in Figure 1. Participants with middle and high father education were more likely to engage in 60-min MVPA than children with low father education (Whole week OR = 1.50, 95% CI = 1.04–2.16, OR = 1.74, 95% CI = 1.09–2.78; Weekend OR = 1.34, 95% CI = 1.05–1.72, OR = 1.43, 95% CI = 1.02–1.99), respectively. Participants with high perceived family wealth were 2.09 [95% CI 1.19–3.66] and 1.81 [95% CI 1.27–2.59] times more likely to meet the PA guidelines than participants with low perceived family wealth on whole week and weekdays, respectively. Participants with middle and high FAS are more likely to engage in 60-min MVPA than children with low FAS (Whole week OR = 1.99, 95% CI = 1.06–3.72, OR = 2.80, 95% CI = 1.48–5.28; Weekend OR = 1.58, 95% CI = 1.11–2.25, OR = 2.60, 95% CI = 1.80–3.75), respectively.

**Figure 2:**
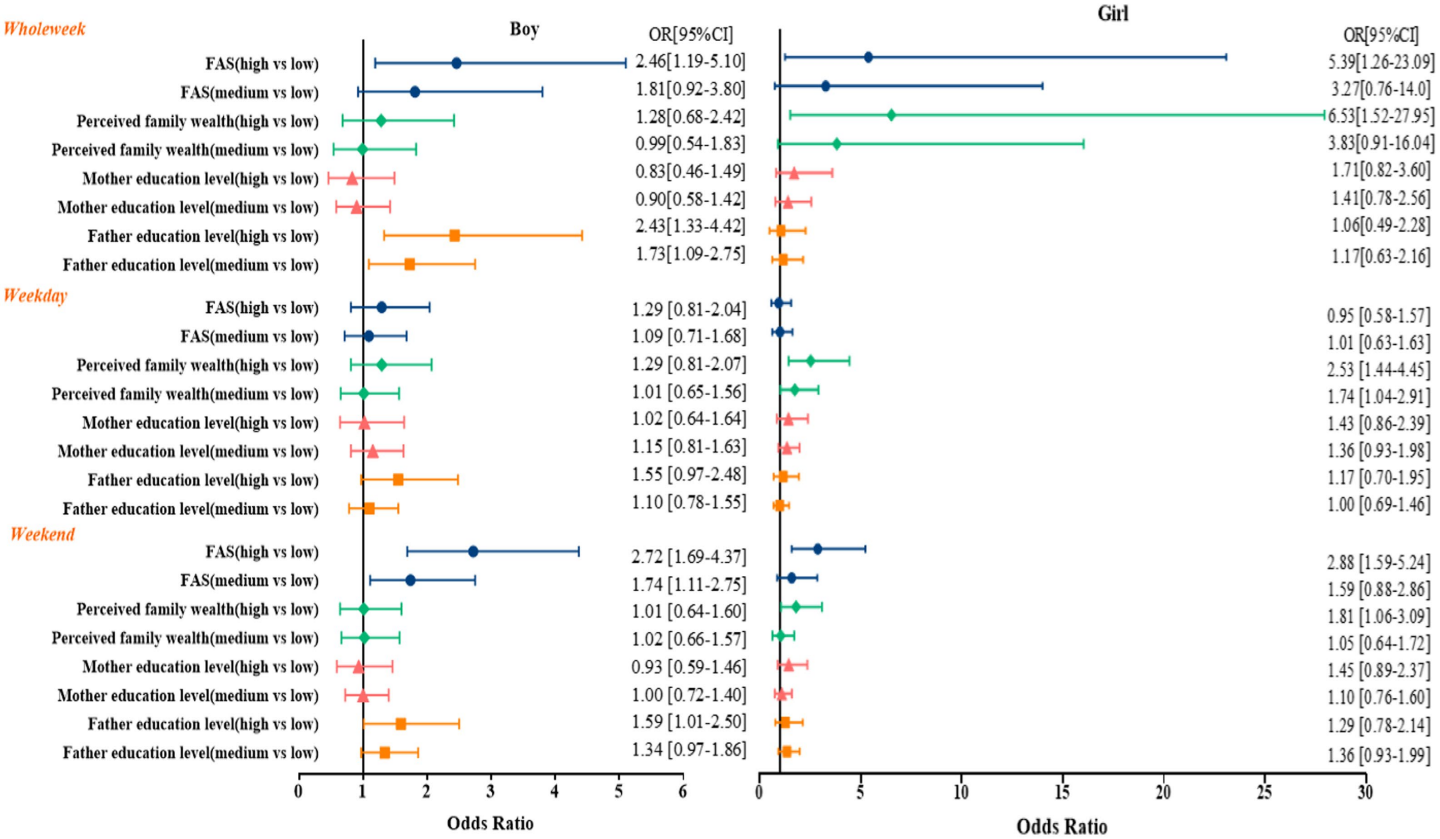
Regression analysis of gender differences in socioeconomic status and physical activity.

The summarized results for the OR for participants meeting the PA guidelines by sex are shown in Figure 2. Boys with high father education levels were 2.43 [95% CI 1.33–4.42] and 1.59 [95% CI 1.01–2.50] times more likely to meet the PA guidelines than participants whose fathers had low education levels on whole week and weekends, respectively. Girls with high perceived family wealth were 2.53 [95% CI 1.44–4.45] and 1.81 [95% CI 1.06–3.09] times more likely to engage in 60-min MVPA on weekdays and on weekends, respectively. Boys and girls with high FAS are more likely to meet the PA guidelines than participants with low FAS on whole week and weekends (Boys OR = 2.46, 95% CI = 1.19–5.10, OR = 2.72, 95% CI = 1.69–4.37; Girls OR = 5.39, 95% CI = 1.26–23.09, OR = 2.88, 95% CI = 1.59–5.24).

**Figure 3:**
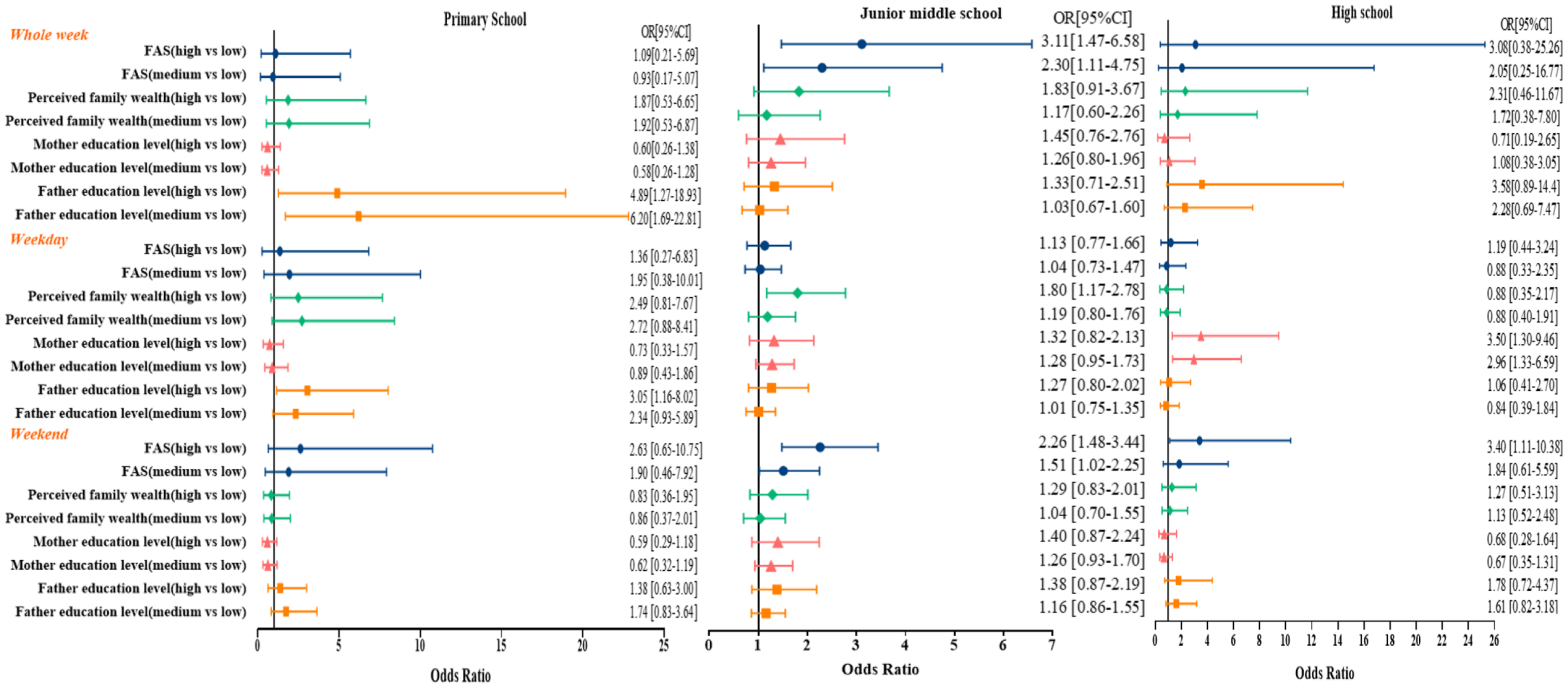
Regression analysis of grade differences in socioeconomic status and physical activity.

The summarized results for the OR for participants meeting the PA guidelines by grade are shown in Figure 3. Primary school students with high father education levels were 4.89 [95% CI 1.27–18.93] and 3.05 [95% CI 1.16–8.02] times more likely to meet the PA guidelines than students whose fathers had low education levels on whole week and weekdays, respectively. Junior high school students with high perceived family wealth were 1.80 [95% CI 1.17–2.78] times more likely to engage in 60-min MVPA on weekdays. Junior high school students with middle and high FAS were more likely to engage in 60-min MVPA than adolescents with low FAS (Whole week OR = 2.30, 95% CI = 1.11–4.75, OR = 3.11, 95% CI = 1.47–6.58; Weekend OR = 1.51, 95% CI = 1.02–2.25, OR = 2.26, 95% CI = 1.48–3.44), respectively. High school students with middle and high mothers’ education level were 2.96 [95% CI 1.33–6.59] and 3.05 [95% CI 1.30–9.46] times more likely to meet the PA guidelines than adolescents whose mothers had low education levels on weekdays, respectively. High school students with high FAS were 3.40 [95% CI 1.11–10.38] times more likely to engage in 60-min MVPA on weekdays.

## Discussion

Although a lot of research have been conducted on the relationship between SES and physical activity among children and adolescents, research in developing countries about this relationship during the past few decades is very scarce (Telama et al., 2009). The results in this study are in line with previous findings demonstrating that SES has an important influence on PA in children and adolescents (Lampinen et al., 2017). In addition, our study gives new insight into the association between SES indicators and MVPA have gender and grade differences, especially on weekends. Judging from the various indicators for evaluating the socioeconomic status, parents’ education, perceived family wealth, and FAS all show their impact on children and adolescents in the same direction.

Our studies have shown that children and adolescents in the mid-level and high-level fathers’ education group are more likely to meet PA guidelines during the week and weekend than in the low-level father’s education group, and the level of the father’s education has no significant effect on the PA of children and adolescents on weekdays. People with high education levels have a better understanding of value of PA and healthy lifestyles, which focused more on participating in exercise in their leisure time, and often ask children to exercise together to improve the level of physical activity in children and adolescents (Finger et al., 2012). However, individuals with lower education levels are mainly engaged in irregular physical work, and probably choose to use media and other entertainment methods to relax during leisure time, which affects their children’s awareness and intention toward physical exercise and have less opportunities and interests in amateur sporting pursuits (Lampinen et al., 2017).Our studies have shown that mother’s education level had no significant effect on PA of children and adolescents during the week, weekdays and weekends. Due to the distribution of the proportion of the test population and other reasons, maybe there is has a significant impact on the MVPA of students of different genders and ages. Previous studies have shown that mothers with low education levels and their offspring are disadvantaged in all aspects of health care, environmental exposure, and health behavior (Gordon-Larsen et al., 2000). Studies have also shown that mother education levels was negatively correlated with MVPA of children and adolescents (Cheung, 2017). However, cultural differences in different regions and differences in measurement methods should be taken into account.

Perceived family wealth is a self-evaluation of children and adolescents on their family’s economic status, which to a certain extent represents a perception of children and adolescents on SES. Low SES families are more concerned about the safety of their neighbors and have fewer physical activity resources available (Estabrooks et al., 2003). However, high SES families are more likely to be a convenient play spaces, good sports equipment, and the easy availability of transportation to sports, and more likely to receive encouragement and support from peers (Qiu et al., 2021). Furthermore, families with higher family wealth can provide more sufficient funds for their children on weekends and even set an example for their children to participate in sports (Mo et al., 2005). Families with lower family wealth cannot fully meet the material basis of children and adolescents for physical activities and lack of available sports venues (Gordon-Larsen et al., 2006).Children from a lower SES may also obtain less parental support and encouragement to participate in supervised PA, be less likely to view their parents as role models in PA, and perceive PA less important than those from a higher SES (Liu et al., 2017; Hong et al., 2020). Such information may help target and design more effective interventions aiming to diminish socioeconomic gradient in children and adolescents’ PA.

### Gender differences in the context of SES

Socioeconomic status has differentiated associations with physical activity among boys and girls, and evident gender differences in the impact of different indicators of SES were found in this study. The gender differences in youth sport participation were found in most countries, there was a study showed a trend toward increased physical activity in girls with higher SES, whereas in boys no significant association occurred between physical activity and SES (Aaron et al., 1993). However, research on an adolescent sample showed that no evidence regarding the influence of socioeconomic status in both genders (Donnelly et al., 2021). The findings of this study suggest that the levels of fathers’ education are positively correlated with boys’ physical activity on weekends, but do not correlate with girls’ physical activity, thus leading to gender inequality in PA promotion. It seems possible that the difference of fathers’ education level may be related to their different attitudes and expectations toward their children’s participation in PA (Schuz et al., 2017). The possible reason for the gender difference is that boys may highly enjoy participating in physical activities and games (Seabra et al., 2013a) and perceive themselves as more physically competent than girls (Qiu et al., 2021). Furthermore, fathers may be more restrictive for vigorous PA in girls than in boys due to cultural conservations (Gustafson and Rhodes, 2006). On the other hand, fathers’ social status seems to be a significant influence on younger boys’ participation in sport (Yang et al., 1996), which in turn makes boys are more susceptible to socioeconomic status, and boys are more likely to receive family economic support in PA (Sallis et al., 1992).

From the perspective of the impact of mother education level, our research showed that mother education level had no significant effect on the physical activity on both, which is inconsistent from previous results (Lampinen et al., 2017). Cultural and lifestyle differences between developed and developing countries might help to explain these differences. Chinese parents may have similar care for their children in family support like in the West, but they may take a different approach to their children’s PA behavior using different strategies. Our research showed that perceived family wealth has no obvious influence on the physical activity in boys but has a noticeable influence on the physical activity in girls on weekdays, which is different from the influence of father’s education level. The evidence showed that girls with a lower SES have been reported to enjoy exertional aspects of exercise less than girls from a higher SES (Seabra et al., 2013a). At present, studies have generally found that there are gender differences in the impact of socioeconomic status on the physical activity of children and adolescents. Previous studies have shown that boys with higher socioeconomic status are more active (Lampinen et al., 2017). However, studies have also found that socioeconomic status does not affect male and female physical activity (Donnelly et al., 2021). The influence of socioeconomic status on the physical activity of boys and girls presents ambiguous results, and this phenomenon is related to the social development of children and adolescents and traditional educational concepts. In addition, the self-efficacy between boys and girls seems to play an essential role in explaining SES differences in adolescent PA (Bauman et al., 2012). Self-efficacy was found to be positively associated with physical activity (Motl et al., 2002; Klazine et al., 2007), and boys have a higher sense of self-efficacy than girls (Seabra et al., 2013b).

FAS has a positive correlation and a significant impact on the MVPA of boys and girls during weekends but has no significant effect on the MVPA on weekdays. Children and adolescents with higher family wealth are more likely to actively engage in sports-club activity on weekends (Pieter et al., 2016),while children with lower family wealth may not be able to access as many extracurricular activities due to financial barriers (Richter et al., 2009).Overall, the effects of FAS on physical activity in boys and girls were relatively straightforward and extensive. In addition, this suggests that the difference in PA between boys and girls caused by SES is more pronounced on weekends, and the difference in PA during school is relatively small. Therefore, more attention should be paid to students’ PA inequalities caused by SES during the weekend.

### School grades differences in the context of SES

The results show that different socioeconomic indicators affect PA of students in different grade groups, especially on weekends. In primary school, students in the ignorant stage have poor independence and autonomy. In addition, the safety of social neighbors also affects the degree of pupils’ physical activities (Voorhees et al., 2009). Junior high school students gradually have their ideas and hobbies when playing sports (Santos et al., 2004). The influence of their parents is gradually reduced, but material wealth has more influence on junior high school students. In the high school stage, they might be expected to be more independent of their family than younger adolescents. Students’ autonomy is improved, and the time spent with their parents is limited. During this period, the impact of parents is relatively weak (Konharn et al., 2013), and more of it comes from the influence of school and peers(Hong et al., 2020; Qiu et al., 2021). The FAS and the mother’s academic qualifications still have a significant impact on the PA of high school students. This may be because, while older adolescents are more socially and psychologically independent from their families than younger adolescents, it may be more expensive for them to participate in sports than younger adolescents, resulting in older adolescents being financially persistent dependent family (Telama et al., 2009).

At the same time, self-determination theory and evidence on the crucial role of integrated and identified motivational regulations for PA (Teixeira et al., 2012). It is important to note that PA behavior may also affect motivational mediation cyclically (Hankonen et al., 2017). Future research should investigate differences in determinants of SES across age groups to understand if and how their roles change throughout life. This study suggests that interventions should target middle school students for socioeconomic disparities in health inequalities. At the same time, it should be noted that the differences in physical activity caused by different socioeconomic status are more pronounced on weekends, indicating that socioeconomic disparities in physical activity among children and adolescents can be relatively reduced during weekdays. Thus, interventions to promote PA related to SES may be more effective on weekends.

### Strengths and limitations

The study extends research on children and adolescents by presenting data from China. This study contributes to the extant research base by exploring the impact of different SES indicators on PA across weekdays and weekends. These findings add valuable knowledge and help inform future efforts to increase PA for children and adolescences. However, the limitations of the study should be recognized. Firstly, cross-sectional design may not satisfactorily explain the causality. Longitudinal studies are needed to provide a better understanding of the causal relationship between SES and physical activity during children and adolescents, which could contribute to a higher success-rate of interventions. Second, PA was not measured objectively. Self-report of children and adolescents may lead to biased results through misreporting, especially for young students. Future studies should use accelerometers or other means to objectively measure PA. Third, the sample is relatively small, involving few regions, and is not nationally representative, a more national representative sample would be desirable. It is important for future studies to apply similar methods across larger national areas.

## Conclusion

In summary, the results show that low SES children are likely to display lower physical activity levels, and there are differences in gender and grade in the impact of different socioeconomic status indicators. This study extends research in this area indicating that potential moderating factors such as SES should be considered in future studies regarding influences of adolescents’ PA levels. At the same time, different socioeconomic status indicators have different effects on students of different genders and different age groups. FAS is more sensitive to physical activity of children and adolescents on weekdays. And socioeconomic disparities in physical activity are relatively weaker on weekdays. It is vital that public health practitioners and healthcare policymakers have access to this important evidence to support public health efforts to empower low SES families. In the future, it is necessary to adopt different intervention measures for different genders and different age pairs in China. In addition, future research should increase the observation of sedentary behavior and sleep indicators on the basis of this study, and explore the relationship between 24-h activity behavior and socioeconomic indicators, so as to comprehensively improve the health of children and adolescents.

## Data availability statement

The original contributions presented in the study are included in the article/Supplementary material, further inquiries can be directed to the corresponding author.

## Ethics statement

The studies involving human participants were reviewed and approved by the Institutional Review Board (IRB) of the Shanghai University of Sport (SUS). Written informed consent to participate in this study was provided by the participants' legal guardian/next of kin.

## Author contributions

YK contributes to the research design, data collection and analyses, and writing. LS, LP, SC, and JH gave many useful suggestions during the writing process. YL supervised drafting of the manuscript, and reviewed it for important intellectual content. All authors contributed to the article and approved the submitted version.

## Funding

This study was supported by grants from the General Project of the National Social Science Foundation of China (19BTY077), the General Program in Education of Planning Project by Shanghai Philosophy and Social Science (A1904) and Shanghai Key Laboratory of Human Performance (Shanghai University of Sport, No. 11DZ2261100).

## Conflict of interest

The authors declare that the research was conducted in the absence of any commercial or financial relationships that could be construed as a potential conflict of interest.

## Publisher’s note

All claims expressed in this article are solely those of the authors and do not necessarily represent those of their affiliated organizations, or those of the publisher, the editors and the reviewers. Any product that may be evaluated in this article, or claim that may be made by its manufacturer, is not guaranteed or endorsed by the publisher.
