# Supplementary material for: Associations between socioeconomic status and physical activity: A cross-sectional analysis of Chinese children and adolescents
Source: Front Psychol. 2022 Sep 1;13:904506. doi: 10.3389/fpsyg.2022.904506 (PMC9477139; doi:10.3389/fpsyg.2022.904506)
Supplement: Supplementary file 1 [file Data_Sheet_1.PDF]

Table 1 Participant characteristics

Category	Boys		Girls		Total	
	n	%	n	%	n	%
Grade						
Primary school	269	51.0	258	49.0	527	17.9
Junior middle	934	51.6	875	48.4	1809	61.2
High school	175	28.3	444	71.7	619	20.9
Total	1378	46.6	1577	53.4	2955	100.0

Table2 Characteristics of Moderate-to-Vigorous Physical Activity

Category	Total MVPA		Weekday MVPA		Weekend MVPA	
	Not Meet (%)	*Meet (%)*	*Not Meet (%)*	*Meet (%)*	*Not Meet (%)*	*Meet (%)*
Total	89.6	10.4	77.7	22.3	74.9	25.1
Gender						
Boys	85.9	14.1	73.2	26.8	69.0	31.0
Girls	92.8	7.2	81.5	18.5	80.0	20.0
P	<0.001		<0.001		<0.001	
Grade						
Primary school	80.5	19.5	70.8	29.2	54.8	45.2
Junior middle school	90.6	9.4	76.2	23.8	76.7	23.3
High school	94.5	5.5	87.7	12.3	86.6	13.4
P	<0.001		<0.001		<0.001	

Table 3 Characteristics of Socioeconomic Status

Category	Father education level			Mother education level			Perceived family wealth			FAS		
	Low	Medium	High	Low	Medium	High	Low	Medium	High	Low	Medium	High
Total	41.0	34.6	24.5	47.7	30.4	21.9	11.3	57.5	31.2	12.8	40.9	46.3
Gender												
Boys	39.6	35.1	25.3	45.6	31.4	22.9	10.4	55.2	34.5	13.1	40.1	46.7
Girls	42.2	34.1	23.7	49.5	29.5	21.0	12.0	59.5	28.4	12.5	41.5	46.0
P		0.345			0.113			<0.05			0.710	
Grade												
Primary	9.1	34.7	56.2	12.5	34.0	53.5	4.9	35.5	59.6	1.9	22.4	75.7
Junior middle school	52.1	33.1	14.8	60.3	27.2	12.5	11.6	62.0	26.5	16.4	44.8	38.8
High school	35.5	38.8	25.7	40.9	36.8	22.3	15.8	63.2	21.0	11.5	45.1	43.5
P		<0.001			<0.001			<0.001			<0.001	
